# Florida Disasters and Chronic Disease Conditions

**Published:** 2006-03-15

**Authors:** Janet Baggett

**Affiliations:** Bureau of Chronic Disease Prevention and Health Promotion, Florida Department of Health

## To the Editor:

Florida had five hurricanes during 51 days beginning on August 12, 2004. More than 8 million residents lost power; more than 9 million people were evacuated; 368,438 people were housed in general and special-needs shelters; and 117 people died. There were long lines for water and gasoline, many homes and businesses were destroyed, and familiar landmarks disappeared. Not broadly publicized was the way people with chronic diseases were affected and how disaster planning will change because of it.

I observed firsthand the obstacles faced by evacuees in special-needs shelters and the impact on Florida Department of Health staff who cared for these people. Florida citizens and leaders have now seen the toll chronic disease takes on quality of life and its impact on disaster relief.

The following information provides a glimpse into the needs of people with chronic diseases during disasters and suggestions for future disaster planning:

- **Designated special-needs shelters.** Florida provides special-needs shelters to meet the needs of people with chronic diseases and disabilities during times of disaster. Each shelter has health professional staff, including at least one physician. Many people arriving at the shelters during recent disasters had diabetes, heart disease, kidney disease, cancer, chronic obstructive pulmonary disease, arthritis, asthma, emphysema, Alzheimer's disease, anxiety disorders, Crohn's disease, cystic fibrosis, depression, epilepsy, multiple sclerosis, Parkinson's disease, or combinations of these conditions. Most people in these shelters needed oxygen, special diets, and medication. Many were unable to make their way to the bathroom without help, could not sleep lying flat, or could not breathe well without oxygen.
- **Shelter beds.** A number of people at our shelters were overweight or obese. Military cots that are normally used for shelter beds were not strong enough and sometimes broke under the excess weight. Getting people on and off of cots was difficult because the cots were so close to the ground. Shelter residents could not maneuver on and off the cots by themselves, and staff members were physically stressed by helping to lift heavy people. The cots also had sharp metal edges that caused many scrapes and bruises — extremely dangerous to the large number of elderly people with thinning skin, to people with diabetes for whom skin integrity is a serious concern, and to individuals taking blood-thinning medication. At the West Florida special-needs shelter where I was assigned to work, we found that chaise lounges were more useful. Other areas of the state reported successfully using reclining chairs instead of beds.
- **Shelter electricity, supplies, and transportation.** Designated shelters benefit by having generators that are located in nonflood areas and that are regularly serviced and tested to ensure that they will work when needed. Shelters need to provide a stockpile of hand sanitizer, gloves, brooms, buckets, disposable wipes, and disinfectant spray to help with the immediate need for infection control. In addition, shelters should have on-site an easy-to-locate, spare key for janitorial and supply closets. The West Florida special-needs shelter had an on-site ambulance and a team of law enforcement and emergency staff. The ambulance and team provided extra security, the ability to move large people, and transportation to the hospital.
- **Diet, nutrition, and food safety.** Many people in our special-needs shelter required special diets. A heart-healthy, low-sodium, low-fat food selection should be standard fare in shelters. Providing such fare may best be accomplished by using prepared meals similar to the military's ready-to-eat meals. In addition, when people are confined, it becomes important to observe precautions for preventing communicable diseases, especially for people with compromised immune systems. Disaster-relief workers would benefit from receiving sanitation, hand-washing, and food-handling education before assignment.
- **Medication.** People often arrived at our shelters without vital medications. Others depleted their prescriptions before they could return home. Residents should continue to be encouraged to bring a list of all prescriptions and the containers for all prescription medications or a copy of the prescription with them to a shelter.  Each state would benefit from having a disaster prescription plan to accommodate emergency prescribing and dispensing by shelter physicians. In addition, the state would benefit by partnering with state, federal, or private prescription insurance providers to identify alternatives for increasing supplies of medications or replacement medications before or during times of disaster.
- **Personal information.** People in our shelters were often unable to recall personal, medical, and insurance information. Severe stress prevented some from recalling the names, addresses, and telephone numbers of next of kin. Individuals should maintain a medical history and insurance card for use in times of emergency. This information would enable health care professionals at the shelter to assess needs.
- **Mass transportation.**  Many low-income and elderly people and people with disabilities caused by chronic diseases need help in getting out of harm's way before disaster strikes. A mass transportation system to move many people over a short time span is preferable. Military buses and helicopters were used in Florida to move some staff in and out of disaster areas. This may become the standard for moving residents away from affected areas. Standing arrangements with nearby states to house evacuees, with a well-defined notification and evacuation plan, is also optimal.
- **Communication.** All modes of communication are used to educate people who may be affected by the storm, especially people with chronic diseases requiring medications and other medical supplies. Materials written in many languages and at a low literacy level are best for educating the widest segment of the population. Florida did an excellent job of educating residents. The state broadcasted public service announcements and distributed written directives.  Public service announcements are available from the Florida Department of Health: http://www.doh.state.fl.us/Hurricane/ Hurricanefactsheet.html.
- **Postdisaster help.** Individuals with chronic diseases, the elderly, and low-income people need help cleaning up their homes when they return from the shelters. Florida residents returned to debris in homes and yards and rotting food in refrigerators and freezers. Many could not tolerate using disinfectant products, did not have the strength or stamina to clean, or did not have the money to hire someone to clean or repair their home. A community-based system of home inspection to determine habitability of homes before people with special needs return may be considered.

The 2004 and 2005 hurricane seasons left many communities without grocery stores, physicians' offices, drug stores, or religious institutions. The entire infrastructure of some communities disappeared entirely. People returning to these communities were stunned and did not know where to turn. Providing help to these people is critical, especially when they are elderly, have a limited income, or have a chronic disease or disability. Florida has developed extensive plans, formed additional partnerships, and collected data on disaster situations that are available to help other states.

This information is a personal account and intended to provide insight into the scope of chronic disease issues that must be addressed during times of disaster. Florida has made many advances in disaster preparation and relief and is ready to help others learn from its experiences.

This was the first time in my 28 years with the Florida Department of Health that I participated in disaster relief at the community level. I am extremely proud of my department and my coworkers for their kindness, courtesy, sympathy, and helpfulness to people affected by these storms. Many worked 24 hours each day for several days in a row and worked through the disaster not knowing the status of their own homes or families. It truly brought out the best in the Florida public health work force and gave the people of Florida a sample of the importance of their public health infrastructure. I hope the lessons learned and the opportunities for improvement that have been identified are acted upon in every state for the benefit of those affected by future disasters.

